# Cupping in Anthrax

**Published:** 1879-04

**Authors:** H. Hunt

**Affiliations:** Beloit, Wis.


### ARTICLE VI.

*Cupping in Anthrax.*

In the early period of my practice, some 40 years ago, I used the cups in the treatment of local diseases more often than now.

During this period I had to treat a bad case of carbuncle, situated on the back of the neck of an old man. While dressing it one day, it struck me forcibly that cupping would be just the treatment for this case.

Calling for a large goblet and some cotton, I applied it as a cup, after expanding the air by burning cotton in it. The effects were truly wonderful—drawing out from the very interior of the tumor a large amount of pus and corruption, which gave immediate relief. The night following, the old gentleman rested for the first time.

Since this experiment, the first one of which I ever heard or knew, I have relied mainly on the cups for the local treatment of carbuncle.

It fulfills the most important indications in the local treatment of this often troublesome and sometimes dangerous disease. It relieves tension and pain, and limits gangrene of the cellular tissue. It materially shortens the time of cure. With appropriate general treatment, the disease is thus shorn of half its pain duration and danger.

The cups may be applied once or twice a day or even oftener. If resorted to in the early stage, the scalpel or lancet should be used to induce a free flow of blood. Mere dry cupping at this time would increase the flow of blood to the tumor without relief. I would caution against too severe cupping until pus is formed

I more often use a large, blunt rimmed tumbler or goblet than any other kind of cup. The size of the opening of the cup should be, if possible, sufficiently large to cover the base of the tumor. An air-pump attached to the cup, if at hand, would be much more manageable and convenient, but the tumbler and cotton may be used with almost equally good effect, if adroitly done, besides having this advantage, of being always available.

H. Hunt, M.D.

Beloit, Wis., Feb. 15, 1879.

